# Lead toxicity in plants: mechanistic insights into toxicity, physiological responses of plants and mitigation strategies

**DOI:** 10.1080/15592324.2024.2365576

**Published:** 2024-06-20

**Authors:** Minoti Gupta, Vinay Dwivedi, Swatantar Kumar, Ashish Patel, Parwiz Niazi, Virendra Kumar Yadav

**Affiliations:** a Department of Biotechnology, University Institute of Biotechnology, Chandigarh University, Chandigarh, Punjab, India; b Amity Institute of Biotechnology, Amity University, Gwalior, Madhya Pradesh, India; c Department of Biotechnology Engineering and Food Technology, Chandigarh University, Chandigarh, Punjab, India; d Department of Life Sciences, Hemchandracharya North Gujarat University, Patan, Gujarat, India; e Department of Biology, Faculty of Education, Kandahar University, Kandahar, Afghanistan; f Department of Plant Protection, Faculty of Agriculture, EGE University, İzmir, Turkey

**Keywords:** Heavy metals, phytostabilization, oxidative stress, phytomining, phytoextraction, bioremediation

## Abstract

Soil toxicity is a major environmental issue that leads to numerous harmful effects on plants and human beings. Every year a huge amount of Pb is dumped into the environment either from natural sources or anthropogenically. Being a heavy metal it is highly toxic and non-biodegradable but remains in the environment for a long time. It is considered a neurotoxic and exerts harmful effects on living beings. In the present review article, investigators have emphasized the side effects of Pb on the plants. Further, the authors have focused on the various sources of Pb in the environment. Investigators have emphasized the various responses including molecular, biochemical, and morphological of plants to the toxic levels of Pb. Further emphasis was given to the effect of elevated levels of Pb on the microbial population in the rhizospheres. Further, emphasized the various remediation strategies for the Pb removal from the soil and water sources.

## 1. Introduction

Due to the rapid increase in population, a drastic increase in industrialization and urbanization has led to substantial growth in pollutants globally.^1–3,3^ Heavy metals (HMs) like, Pb, Cd, Cu, Hg, As, Co, Ni, Mo, etc. are some of the most prevalent contaminants found within the environment whose sources are mainly industries, automobiles, etc.^4^ Out of these HMs, some are less toxic while some are highly toxic to environment and the living beings.^5,6^ The HMs (Pb, Cd, As, Cu, and Hg) become a potential environmental hazard once their concentrations reach a toxic level.^7,8^ These HMs have a major role in altering the pH of the soil, making it unsuitable for the growth of the plant.^9,10^ These toxic HMs could originate from either natural sources, for instance, volcanic eruptions, or anthropogenic activities like smelting.^11^

**Figure 1.**
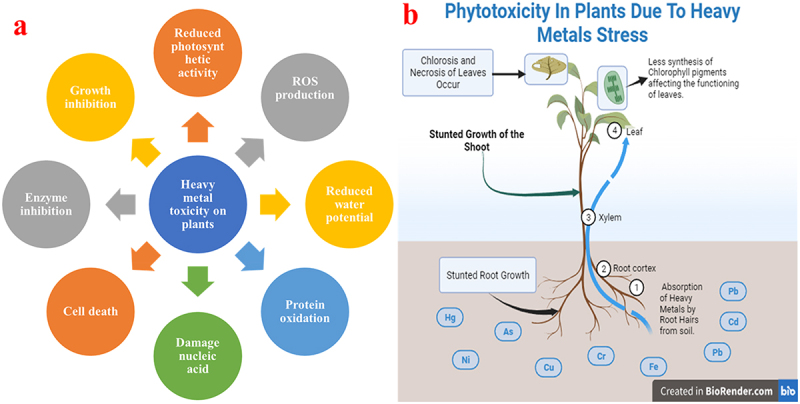
a) Adverse effects of heavy metals on plants, b) phytotoxicity in plants due to the HM stress.

From the various pieces of literature, it has been evident that HMs may exert various effects on plants which may cause a major challenge to farming, ecosystem health, and human beings.^12^ Among all the HMs Pb, Cd, and As, could disrupt cellular structures, hamper important metabolic activities, and trigger oxidative stress (OS).^13^ The HMs toxicity may cause hindered plant growth, undeveloped root and shoot, chlorosis, necrosis, and ultimately less plant mass.^14^ Firstly, there is a possibility of entering these toxic HMs into the edible parts of the plants, from which they may enter the human food chain, threatening food safety and human health.^15^ Understanding the side effects of HMs on plants for developing efficient approaches is very important to mitigate their consequences on the environment and human health.^16,17^ More research along with detailed insights is required on HMs and Pb-based toxicity in plants. Figure 1a shows the adverse effects of HMs on plants, while Figure 1b shows phytotoxicity in plants due to the HM stress.

Pb is one of the major global concerns due to its toxicity to the ecosystem and living beings.^18^ The global production of Pb is continuously increasing due to its high demand in batteries, automobiles, and other industries.^19^ Pb is considered one of the most hazardous HMs which severely affects plant life resulting in decreased soil fertility.^20^ It is a non-essential HMs, introduced into the environment by numerous natural and anthropogenic activities.^21,22^ It remains persistent in the environment (soil, water, and air) which poses a potential threat to the ecosystem.^23,24^

The Pb toxicity in the soil significantly alters the diversity and metabolic functions of the microbial population which further minimizes microbial biomass and enzymatic activities important for the health of the soil.^25^ As a result, there will be a reduced fertility of the soil eventually affecting the plant growth and agricultural productivity.^26^ The Pb toxicity further depends on the pH, alkalinity, and solubility.^27^ There is a possibility of bioaccumulation and biomagnification of the Pb at each level of the food chain, which further aggravates its ecological impact, threatening the health of higher trophic-level organisms.^28^ The germination of seeds, growth, and metabolic activities of plants are affected by the increased level of Pb, resulting in decreased crop yields.^29^ Furthermore, due to HMs stress, there is a generation of OS in the plants which are responsible for the excessive production of reactive oxygen species (ROS) [OH^−^, O_2_^2−^, H_2_O_2_] that causes damage to the plants at the cellular level.^30^

There is a requirement to mitigate Pb contamination in the environment by formulating eco-technological solutions like chelation therapy and biological remediation methods for detoxifying soils. To address the Pb contamination there is a need for global cooperation to reduce industrial emissions, improve waste management, and phase out Pb-containing products.^31^

It has been found that in a Pb-contaminated environment, the plant develops a well-developed defense mechanism (utilization of enzymatic and non-enzymatic antioxidants) to overcome stress induced by Pb toxicity.^1^ It involves a network of enzymes, organelles, and signaling pathways that team up to lower the damage caused by ROS.^32^

Recently toxicity of Pb in plants has been reported by several investigators for instance Zulfiqar et al., highlighted the impact of toxicity of Pb in plants and the strategies developed by plants for remediation of Pb.^33^ Srivastava and Srivastava (2023) highlighted the molecular mechanism of Pb toxicity in plants and their tolerance.^34^ Pourrut et al., emphasized the mechanism involved in the uptake and detoxification of Pb in higher plants at the molecular level.^35^ Colin et al., emphasized the bioaccumulation of Pb and its effects on plants.^1^ Recently, Martinz et al., performed a case study for the phytoremediation potential of native plant species for Pb-contaminated soils. Moreover, in the same year Kim et al., observed the genetic variation in lead tolerance among plant populations. Further, Zhou et al., performed a meta-analysis study for the effects of toxicity of Pb on photosynthesis and chlorophyll fluorescence parameters in plants.

In the present review, investigators have emphasized lead toxicity in plants, their responses at different levels, and strategies to remediate Pb toxicity in plants. The investigators have emphasized the increasing global concern of Pb in the environment. Emphasis has been given to the different sources of the Pb in the environment, and their uptake mechanism by the plant from soil. Emphasis has also been given to the various Pb adaptive responses in the plants at the physiological, and molecular levels. Investigators further emphasized the various changes in the plants due to Pb stress. Finally, investigators highlighted the remediation strategies for Pb toxicity in plants and the environment.

## 2. Sources of Pb in the environment

Pb is present naturally in the environment and it is not required by plants for their growth. The sources of Pb could be either natural or anthropogenic.^36^ Naturally, it is present in the geological samples, which enter to environment via volcanic eruptions.^37,38^ Anthropogenically, it is released from automobile exhaust, industrial effluents, and Pb-containing fertilizers.^39^ Moreover, it is also present in the batteries which enter into the environment after the dumping of old batteries.^40^

### 2.1. Natural sources

Pb is present in the Earth’s crust naturally and is discharged into the environment through natural phenomena like sea spray emissions, weathering, volcanic eruptions, and the re-release of historical sources like Pb in sediment.^18^ Lead from mining areas and erosion also contribute to its presence in the Earth’s ecosystem. Forest fires and biogenic sources are additional natural contributors to soil pollution. Weathering predominantly facilitates the transfer of lead from natural sources to the air, dust, water, and sediment.^41^

### 2.2. Anthropogenic activities

**Figure 2.**
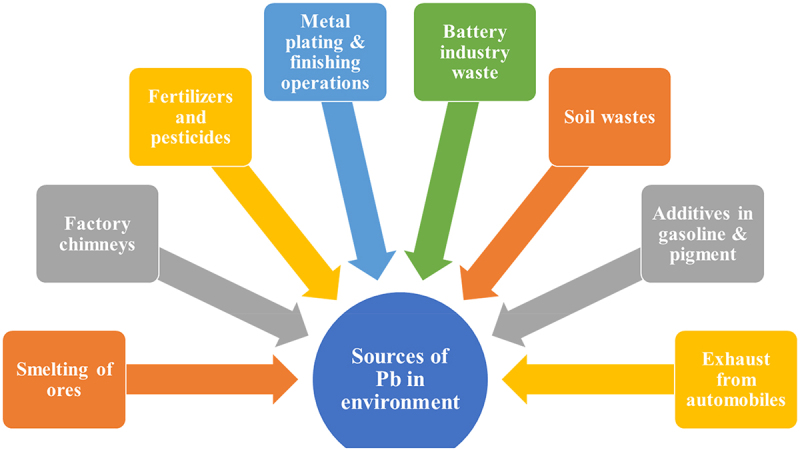
Various sources of Pb in the environment.

About 98% of lead pollution is due to several anthropogenic sources, including paints, petrochemical refineries, lead batteries, leaded gasoline used in motorsports and aircraft, batteries, smelting process of lead ore, toys, paint, gasoline herbal remedies, ceramic materials, and the fertilizer industry, contribute to the contamination of the terrestrial and aquatic ecosystems.^42-46^ The release of toxic Pb from automobile manufacturing and refining refineries is responsible for the contamination of the ecosystem.^47^ Figure 2 describes the various environmental sources of Pb.

## 3. Effect of lead contamination on soil microorganisms and soil health

Pb contamination in soil can have a significant impact on soil microorganisms and soil health, which can in turn negatively affect plant growth and ecosystem functioning.^48^ Being a HM Pb inhibits the potential of various soil microorganisms, including bacteria and fungi, by disrupting their cellular processes and enzyme activities^49^ which results in the lowering of the activity and biomass of microbes, due to which the cycling of nutrients and decomposition of organic matter is disrupted. This, in turn, leads to changes in soil structure, water-holding capacity, and nutrient availability, which negatively affects plant growth and ecosystem functioning.^33^ Various pieces of literature have found that the concentration of Pb in polluted soils ranges from 400 to 800 mg/kg^50^ whose value rose to up to 1000 mg/kg in the industrial area soil. The extent to which Pb is retained in the soil mainly depends on the pH, as acidic soil absorbs more Pb than alkaline soil.^51^ As a result, lead is considered a major ecological pollutant.^52^ The leakage of petroleum hydrocarbon from refineries often introduces Pb into the soil, resulting in decreased soil porosity, microbial biomass and diversity, and microbial activity, which ultimately hinders plant growth.^53^ Moreover, Pb contamination also affects soil enzymatic activity, with some enzymes being inhibited and others being activated, depending on the type of enzyme and the level of Pb contamination.^54^ Overall, Pb contamination in soil may result in significant adverse impacts on soil microbial community and soil health, which can affect plant growth and ecosystem functioning.^55^ Therefore, it is important to implement effective remediation strategies to mitigate these impacts and ensure the health and sustainability of soil ecosystems.

## 4. Lead uptake and accumulation in plants

**Figure 3.**
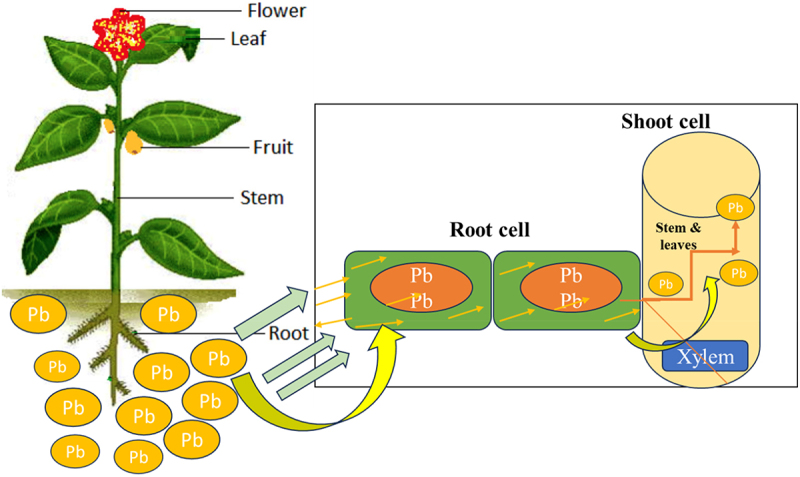
Cellular uptake, transport, and translocation of Pb in plants modified.^60^

Pb being an environmental pollutant is a potential threat to both aquatic and terrestrial ecosystems. It is of utmost importance to understand the phenomena by which plants uptake and accumulate lead is crucial for developing strategies to mitigate its harmful effects and safety of human beings.^56^ The entry of Pb in plants is a critical issue that impacts environmental conservation and agricultural sustainability.^57^ The understanding of the mechanism involved behind Pb absorption and its retention in the plant is very important as thus will help in developing interventions to reduce its harmful effects on ecosystems and food security.^58^ The entry of Pb in plants occurs through root absorption and foliar uptake, which further undergo chemical interactions within the plant that affect its dispersion and toxicity.^36^ Accumulation of Pb in edible parts of the plant poses a significant risk to human health, which may lead to neurological disorders and developmental abnormalities in the long term.^50,59^ Figure 3 demonstrates the uptake by cells, transport, and translocation of Pb in plants.

### 4.1. Mechanisms of Pb uptake by plants

The mechanisms of lead uptake are governed by various environmental factors, like Pb concentration, soil moisture levels, and the presence of competing cations in the soil solution.^36^ The uptake of Pb in plants is due to passive diffusion, facilitated transport, and active uptake. Passive diffusion occurs when Pb ions move across the root cell membranes down their concentration gradient.^61^ Facilitated transport involves the uptake of Pb by specific membrane transport proteins, such as aquaporins and ion channels, which facilitate the movement of Pb across the root cell membrane.^62^ Active uptake mechanisms are primarily facilitated by transporters belonging to the Zinc-Iron Permease (ZIP) and Natural Resistance-Associated Macrophage Protein (NRAMP) families, which enables the plants to actively transport Pb ions against their concentration gradient into root cells.^63,64^

**Figure 4.**
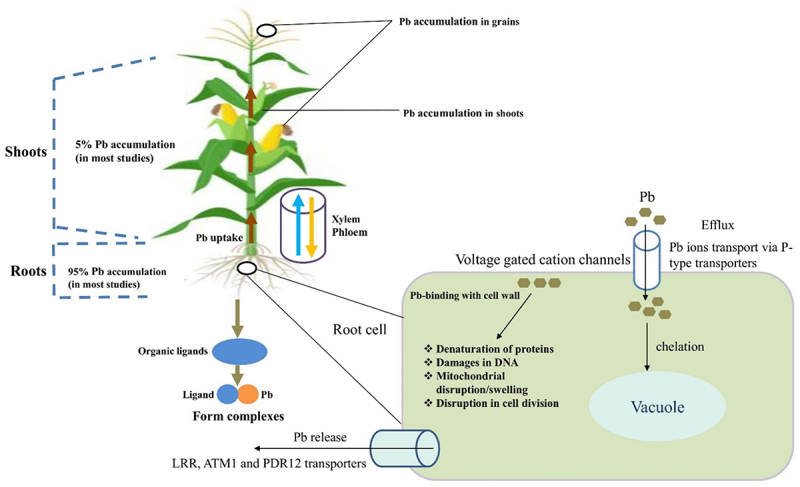
Potential uptake mechanisms and accumulation of lead (Pb) in plant parts.^74^

The roots of the plant absorb the divalent free-Pb^2+^ available in the Pb-contaminated soil, which is subsequently carried via xylem vessels along with other nutrients and unloaded in the endodermis.^65^ The roots accumulate a higher amount of Pb than any other parts (fruits and vegetables) of the plant as roots are in direct contact with the soil and due to restricted translocation caused by factors such as the Casparian strip endodermis.^66,67^ The endodermis acts as a physical barrier, preventing Pb ions from moving through the vacuole of the leaf. The Casparian strip, found in plant root endodermis, acts as a selective barrier to block the movement of Pb from the root to the shoot.^68^ It’s made of a hydrophobic substance called suberin and forces water and nutrients to pass through endodermal cells, allowing selective transport of essential nutrients while blocking harmful substances.^69^ This vital mechanism protects the shoot from Pb toxicity, which enables the plants to thrive in contaminated environments. It helps in reducing Pb transportation through the symplast pathway only, lowering the amount of Pb in the transportation process.^70^ For instance, uptake of Pb could follow the following order: root uptake, foliar absorption, mycorrhizal association, phloem transport, storage and accumulation, and translocation to edible parts.^71^ Roots will first uptake the Pb either by passive diffusion or by active transport. Passive diffusion involves the movement of Pb ions from the soil into the root cells along a concentration gradient. Active transport involves the transportation of Pb ions into the root cells by specialized transport proteins, such as ion channels or pumps.^72^ The foliar absorption of Pb could take place by two means i.e. either cuticular penetration or by stomatal uptake, wherein the former Pb ions are actively transported into the root cells by specialized transport proteins, such as ion channels or pumps. In the latter case, Pb ions get access to the leaf through stomatal openings, where they are subsequently transported to other parts of the plant.^73^ Figure 4, shows the uptake mechanism and accumulation of Pb in parts of the plants

In the mycorrhizal association, the mycorrhizal fungi facilitate the uptake of Pb by developing symbiotic associations with the roots of the plants, which increases the surface area available for Pb absorption and enhances nutrient uptake.^75^ In the phloem transport, once the Pb ions get absorbed by the roots or leaves they get transported throughout the plant via the phloem, allowing for systemic distribution to various tissues and parts.^76^ Further, these Pb ions get stored and accumulated and at this event, the Pb ions may undergo numerous transformations within the plant like binding to organic ligands or sequestration in vacuoles, leading to their accumulation in different plant compartments, including roots, shoots, and fruits.^77^ Finally, the accumulated Pb gets translocated and stored in the edible parts of plants like fruits, and grains which challenges a potential risk to human health after consumption.^78^

Pb accumulation in plants occurs through various processes including absorption, bioavailability, bioconcentration, and biomagnification. With the decrease in pH, there is an increase in the solubility of the because at lower pH the Pb is more soluble in the soil solution.^79^ Although Pb has no biological function in plants, it accumulates mainly in roots, leading to high concentrations in root vegetables such as sweet potatoes and carrots.^66^ As the organs that take up essential elements and HMs from the soil, roots play a central role in the growth of the plant.^5^

### 4.2. Factors influencing Pb accumulation in plants

The accumulation of Pb ions in the plants could be influenced by several factors including plant species, growth stage, tissue type, and the physicochemical features of the soil (pH, organic matter content, and cation exchange capacity) which affect the availability and mobility of Pb ions in the rhizosphere.^36^ Some of the plant species exhibit a higher affinity for the uptake and accumulation of Pb due to differences in root morphology, physiology, and biochemical characteristics.^80^ Moreover, the plant’s growth stage could also affect its susceptibility to lead accumulation for instance younger plants generally accumulate higher concentrations of Pb in comparison to mature plants.^81^ The concentration of the Pb could vary in different parts of the plant tissues for instance roots typically accumulate a higher quantity of Pb than shoots and leaves.^1^ Moreover, environmental factors (soil pH, organic matter content, and redox potential) could also play a major role in determining the bioavailability and mobility of Pb in the soil-plant system, which may further influence Pb accumulation in plant tissues.

It could be concluded that the uptake and accumulation of Pb in plants is a complex phenomenon that is controlled by a combination of physiological, biochemical, and environmental factors.^82^ Understanding the uptake and accumulation of Pb along with the factors involved in the accumulation of Pb in plants could help in developing effective strategies to mitigate contamination of Pb in the environment and reduce its impact on ecosystem health and human welfare.^82^

## 5. Physiological responses of plants to Pb stress

**Figure 5.**
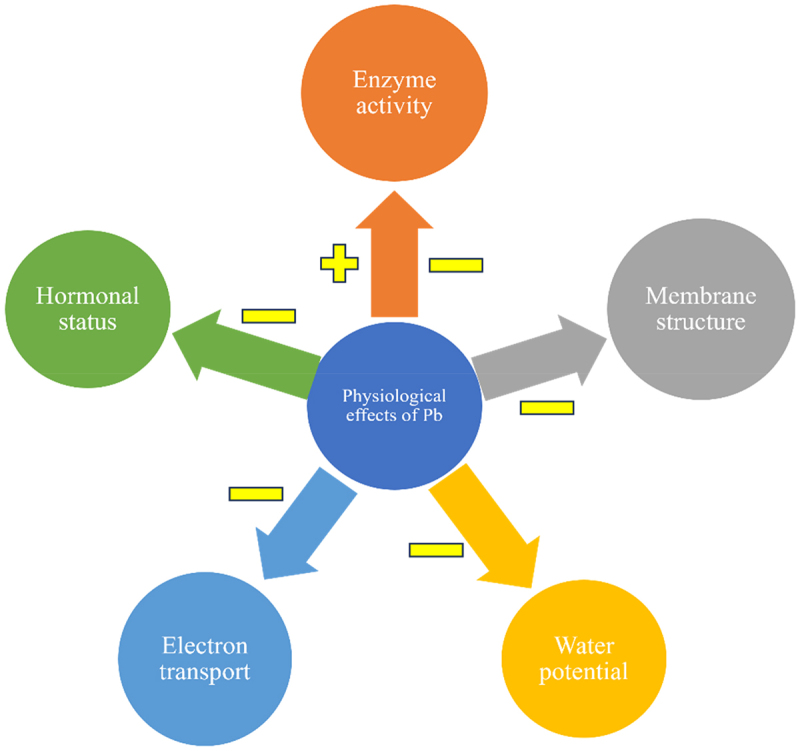
Overall disturbance in physiological mechanisms in plants by Pb toxicity. Here, “+” and “−” signs indicate positive and negative effects.^74^

Pb stress causes overall disturbances in the physiological mechanisms in plants like enzyme activity, membrane structure, water potential, electron transport, and hormonal status. All these fatutes fall I the physiological changes in the plants. Due to Pb stress enzyme activity of the plants was affected both negatively and positively. The membrane structure, water potential, electron transport, and hormonal status are negatively affected by the Pb. Figure 5 exhibits Pb toxicity-based disturbances in the physiological mechanism of the plants.

There are numerous ways by which plants respond to the Pb ions stress which could be expressed in terms of morphological changes, biochemical responses, and molecular responses which are described below in detail.^82^

### 5.1. Morphological changes

The morphological changes in the plant could be used as a visual indicator of plant stress and could provide valuable insights into the extent of Pb contamination in the environment.^83^ Due to the Pb stress, there is an induction of numerous morphological changes in all the parts of the plants like delayed seed germination and reduced seedling growth, stunted root growth and shoot growth, reduced branching of roots, reduced overall root biomass, reduced flower production, photosynthetic pigments, and mineral nutrients.^84^ Among the roots, it has been observed that there is an inhibition of root elongation (due to interference of Pb with the cell division and elongation in root tips) and shorter and thicker roots. Pb-stress may further exhibit chlorosis, necrosis, and leaf curling, in the parts of the plants above the ground. These symptoms suggest reduced photosynthetic efficiency and decreased nutrient uptake.^85^

Pb cannot reach the seed’s developing interior tissues until the testa is broken by the developing radicle.^86^ Comparatively, dicots store more Pb in their roots as an organometallic compound than monocots. Investigations showed that Pb at high concentrations (1 mM) lowered rice germination by 14–30% and stunted seedling growth by more than 13–45%. Moreover, there were decreased germination rates and short hypocotyl and roots in *Lupinus* due to Pb toxicity (Envis Centre on Plants and Pollution, 2021).

A team led by Kanwal reported that lead toxicity has detrimental effects on several aspects of wheat crop growth and quality. These effects encompass germination, physiological processes, biochemical activities, yield, mineral content, and overall growth attributes. The underlying cause of these adverse impacts is a lowering in the rate of photosynthesis, which, in turn, leads to decreased transpiration and stomatal conductance.^87^

### 5.2. Biochemical responses

The enhanced Pb concentration may disrupt natural physiological processes, reduce enzyme activity, interfere with inorganic nutrition and hydration management, and alter the permeability of the cell membrane leading to phytotoxicity, and eventually lead to cell death.^34,88^

The elevated level of Pb in plants leads to numerous biochemical or physiological changes for instance; decreased germination rates, decreases in the production of protein, RNA, and DNA in the embryonic axis and endosperms of plants, and opening and closing of stomata.^77^ Pb decreases the germination rate by interfering with amylase and protease enzyme activity, as well as with metabolic functions, inhibiting the ATPase enzyme, and leading to a decreased energy production in the seed embryo.^34^ There is a lowered transpiration rate due to Pb toxicity due to which the amount of water and nutrients reaching the plants’ upper regions is affected adversely. The decreased production of protein, RNA, and DNA in the embryonic axis and endosperms of plants, results in reduced germination of the seedlings.^1^ Besides this, an increase in electrolyte leakage, malondialdehyde accumulation, non-enzymatic and enzymatic antioxidants, osmolytes, and free radicals (H_2_O_2_, O^2^− occurs due to Pb toxicity in the plants.^33,89,90^ Due to increased free radicals, there will be an altered biochemical structure of macromolecules, which will reduce physiological and biochemical activity and interfere with the functioning of enzymes involved in metabolic processes.^91^ This results in suppressed photosynthetic activity reduced ATP synthesis, and a decrease in the growth and development of the plants.^92^ The accumulation of Pb in soil disrupts water balance, nutrition, mineral and enzyme activity, and increases the level of oxidative damage in plants. Pb enters plant cells by altering the composition of lipids and proteins in the cell membrane.^74^ Furthermore, Pb stress can disrupt ion homeostasis in plants, leading to alterations in nutrient uptake and distribution. For instance, Pb competes with essential cations such as calcium (Ca), magnesium (Mg), and potassium (K) for uptake by root cells, resulting in nutrient imbalances and physiological disorders.^82^

To overcome the toxic effects of Pb, plants employ numerous various defense mechanisms for instance synthesis and accumulation of antioxidants, including superoxide dismutase (SOD), catalase (CAT), and glutathione peroxidase (GPX), which scavenge reactive oxygen species (ROS) generated under lead stress.^93^ Enhanced antioxidant activity helps alleviate oxidative damage to cellular components such as lipids, proteins, and nucleic acids, thereby maintaining cellular homeostasis.^94^ Additionally, plants may change secondary metabolite production, including phytochelatins and metallothioneins, which chelate and sequester lead ions, reducing their availability and toxicity.^95^ Elevated levels of activity of the antioxidant enzyme and gene expression for CAT, SOD, MDA, and GPX were observed in the roots of wheat cultivars in response to elevated lead levels in the soil.^96^

#### 5.2.1. Lipid peroxidation (LiPr)

Occurs due to the damage caused by free radicals to the membrane and the cells they contain.^97^ Biochemical parameters such as antioxidant enzyme activity increase under increased Pb concentration in various plant species.^82^ Due to the increased Pb concentrations, there is an increase in respiration rate in plant cell mitochondria and disturbances in the electron transport chain due to the binding of lead with mitochondrial membranes. Studies by Sidhu et al., and several others like Gupta et al.; Dias et al., (2019); Navabpour *et al*., (2020)^98–102^; have provided insights into the various effects of Pb exposure on plants. However, there is still no clarity on the location of the action of Pb ions or the underlying process that affects photophosphorylation.

#### 5.2.2. Photosynthesis

Photosynthesis is very important for the proper growth and development of plants, and it facilitates normal physiological, biochemical, and molecular activities within plant cells.^103–105^ However, Pb toxicity affects the biosynthesis of chlorophyll, carotenoids, and plastoquinone, as well as reduces the activity of C_3_ cycle enzymes, thereby impeding the rate of photosynthesis. Pb’s affinity for protein *N*- and S-ligands also causes damage to the photosynthetic system.^70^ The elevated level of Pb causes a decline in both photosynthetic and respiratory rates which is due to a reduction in the mitochondrial cristae density, resulting in a reduced rate of oxidative phosphorylation potential.^106^ Pb toxicity leads to reduced water transport from roots to leaves, which is linked to photosynthesis and CO_2_ absorption rates is crucial for cell turgor and plasticity, and is physically impeded by Pb.^70^ The exposure of plants to higher levels of Pb results in an increase in chlorophyllase enzyme activity, which in turn causes an increase in chlorophyll breakdown.^107^ A decline in chlorophyll content has been reported in wheat (*Triticum aestivum L*.) cultivars, Gonbad, Morvarid, and Tirgan, when exposed to enhanced Pb levels in the soil.^99^ Pb treatment has a higher impact on chlorophyll b than chlorophyll a, and it has a profoundly negative effect on the development of α-amino levulinate dehydrogenase, an essential enzyme in the synthesis of chlorophyll.^108^ Lead exposure also affects the cytochrome b6f complex, photosystem I, and sites for donors and recipients of photosystem II. Photosystem I is known to be less vulnerable to lead inhibition than photosystem II.^109^ Lead stress reduces the photocatalytic activity of plants, leading to changes in the morphology of chlorophyll in *Citrus aurantium* L. Chlorophyll b is more severely affected than chlorophyll a due to Pb’s impact on lipid composition, which can also result in a reduction of the lamellar system and the number of starch grains in leaves.^110^ Inhibiting photosynthesis not only affects plant growth but also has a profound effect on the development of fruits and flowers.^89^

#### 5.2.3. Stomata

Moreover, a higher level of Pb damages cellular membranes resulting in stomatal closure, which leads to a deficiency of CO_2_ and disrupted water relations.^33^ The uptake of Pb by plants slows transpiration rates, which stops the flow of water and nutrients from the soil to the plant, thereby suppressing growth and biomass accumulation.^50^

#### 5.2.4. Nutrient

Pb toxicity also results in minimized uptake of essential components such as Mg and Fe, which are essential for chlorophyll production, leading to decreased chlorophyll synthesis.^111^ The Pb toxicity causes a modification in the permeability of the chloroplast membrane, which is caused by an imbalance in the protein and lipid ratio within the plasma membrane of the chloroplast.^112^

**Figure 6.**
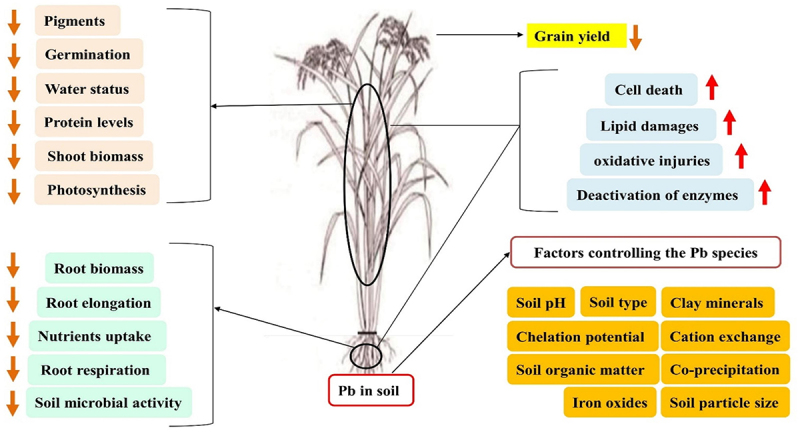
Thematic of Pb stress in plants.^74^

Pirzadah et al., observed that Pb stress affects the defense mechanisms of Tartary buckwheat plants. This influence was visible as there was an increased synthesis of osmolytes and antioxidant enzymes, which appeared to be critical in assisting plants in coping with stressful conditions.^113^ Figure 6 shows the various effects of Pb stresses in plants.

### 5.3. Molecular responses

Due to the Pb stress response, there is an orchestrating of a complicated network of gene regulatory phenomena in the plants at the molecular level.^114^ Transcriptional profiling analysis has demonstrated the differential expression of genes involved in stress perception, signal transduction, and stress tolerance pathways in Pb-stressed plants. These include genes encoding transcription factors (e.g., AP2/ERF, WRKY, NAC), stress-responsive proteins (e.g., dehydrins, LEA proteins), and detoxification enzymes (e.g., metallothioneins, glutathione S-transferases).^115^ Moreover, the Pb stress could trigger the activation of numerous signaling pathways, including MAPKs and calcium-dependent protein kinases (CDPKs), which coordinate downstream stress responses.^116,117^ Additionally, epigenetic modifications (DNA methylation and histone acetylation), also play an important role in the regulation of gene expression under Pb stress environments, which modulates the adaptive potential of plants to manage environmental challenges.^118,119^

The stress induced by Pb is a significant threat to plants in their natural conditions, which causes harm by disrupting their vital cellular activities.^120,121^ For maintaining the plant cell’s physiological and metabolic homeostasis, stress needs to be decreased due to HMs accumulation. MAPKs cascade transmits environmental signals from cell membrane receptors to the nucleus of plant cells through mechanisms like phosphorylation and dephosphorylation.^122^ This cascade targets various effector proteins and TFs, which eventually leads to a stress response in plants. Plant hormones, nitric oxide (NO), and ROS activate the MAPK cascade by influencing gene expression, activating antioxidant systems, and facilitating communication among different signal molecules.^123^ Downstream TFs, regulated by MAPK, enhance plant tolerance and Pb accumulation. The MAPK cascade is well-known for its involvement in plant responses to drought but is less explored in the context of plant reactions to Pb.^122^ Pb is highly toxic, swiftly affecting plant health by causing nutrient deficiencies, inhibiting chlorophyll synthesis, reducing photosynthesis inhibiting growth and plant death.^124^ In response to Pb stress, plant roots initiate various physiological and structural fluctuations. These responses involve changes in gene expression, increased antioxidant activity, and intricate crosstalk between signaling molecules.^125^ Multiple defense responses are activated to improve plant survival in HMs-contaminated environments.

WRKY genes are a family of TFs found in plants which are named after a conserved amino acid sequence WRKYGQK in their DNA-binding domain.^126^ These genes play an important role in regulating several physiological methods in plants, with a significant emphasis on their function in plant stress responses.^127^ WRKY TFs bind to specific DNA sequences known as W-boxes (TTGACC/T). These binding events allow WRKY proteins to regulate the transcription of target genes by either activating or repressing their expression. These genes are involved in plant responses to several biotic (living organisms, such as pathogens) and abiotic (non-living factors, like, drought, salinity, and temperature) stresses. They act as key regulators of stress-responsive genes (SRG). In response to pathogen attacks, WRKY genes play a central role in activating defense mechanisms.^128^ They regulate the expression of genes associated with pathogen recognition, signaling, and defense response, including the production of antimicrobial compounds. WRKY genes are also engaged in plant responses to abiotic stresses.^129^ In a study by Li et al., (2020) on Al-stressed *Arabidopsis thaliana* it was found that WRKY47 acts as an important transcription factor by helping the Al-stressed plants in improving the cell wall remodeling, which increased the growth of the roots and also helped the plants to overcome stress induced due to Al toxicity. ELP and XTH17 the important genes were regulated by WRKY47 and also contributed to the balanced distribution of Al in the root cells.^130^ They can modulate the expression of genes related to stress tolerance mechanisms, such as those involved in drought resistance or the detoxification of HMs. In *Arabidopsis*, WRKY12 inhibits Cadmium accumulation and tolerance by directly targeting GSH1 and indirectly decreasing PC synthesis-related gene expression.^131^ These genes interact with and are regulated by plant hormones, for instance, jasmonic acid (JA), salicylic acid (SA), and ethylene (ET). These hormones are essential for signalling stress responses and can induce the expression of specific WRKY genes. These genes are part of complex signalling networks and crosstalk with other TFs and pathways, including MAPK cascades and other stress-related pathways.^128^ This allows plants to fine-tune their stress responses. The WRKY gene family consists of multiple members (isoforms) with varying DNA-binding specificities and affinities.^132^ Different WRKY isoforms may be activated in response to specific stressors, leading to a tailored stress response. So, the authors can conclude that WRKY genes are key players in plant stress responses, both in defense against pathogens and adaptation to adverse environmental conditions. They regulate the expression of SRG and are essential for a plant’s potential to combat and cope with a wide range of stressors.

NAC genes (NAM, ATAF, and CUC) belong to a large family of plant-specific TFs. These genes are named after the three founding members: NAM (No Apical Meristem), ATAF (Arabidopsis Transcription Activation Factor), and CUC (Cup-Shaped Cotyledon). NAC TFs play versatile roles in plant development and stress responses. NAC genes are crucial regulators of plant development. They control processes like embryogenesis, floral organ formation, lateral root development, and senescence.^133^ NAC TFs are involved in both abiotic (non-living) and biotic (living organisms, such as pathogens) stress responses in plants.^134^ Their roles in stress include:-

- **Abiotic stress**: The activation of NAC genes takes place in response to various environmental stresses, for instance, drought, salinity, cold, and heat which regulate the expression of SRG, contributing to stress tolerance and survival. In Arabidopsis, the NAC gene DREB1A/CBF3 regulates cold stress responses.^135^
- **Biotic stress**: Some NAC genes are implicated in plant defense mechanisms against pathogens which could modulate the expression of genes involved in pathogen recognition and defense responses. NAC TFs may also interact with other defense-related proteins and hormones, like, salicylic acid and jasmonic acid.^135^
- **Senescence regulation**: NAC genes are associated with the regulation of senescence, i.e., control the timing of senescence and leaf death by promoting the expression of senescence-associated genes.^136^
- **Hormone signaling**: NAC genes can interact, and get regulated by plant hormones. These interactions allow them to integrate hormonal signals into stress responses like NAC transcription factor ANAC019 in *Arabidopsis* is induced by abscisic acid (ABA) during drought stress.^137^
- **Diverse targets**: NAC proteins often function as TFs, by binding to specific DNA sequences in the promoters of target genes which can activate or repress the expression of these target genes, depending on the specific NAC gene and its associated target genes.^138^
- **Isoform specificity**: Like other transcription factor families, the NAC gene family comprises multiple members, each with its unique characteristics and functions. Different NAC genes may respond to distinct stressors or developmental processes.^139^

**Figure 7.**
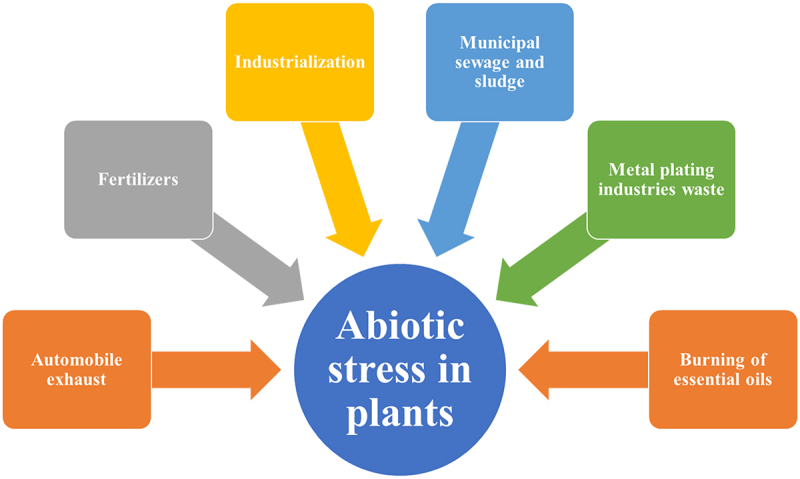
Different abiotic stresses in plants.

So, the authors can conclude that NAC genes play a vital role in plant stress responses, helping plants adapt to a wide range of environmental challenges, from abiotic stressors like drought and temperature extremes to biotic stress from pathogens.^140^ They do so by regulating the expression of genes engaged in stress tolerance and defense mechanisms. Additionally, NAC genes have diverse functions in plant development and senescence, making them key players in the plant’s life cycle. Figure 7 shows various abiotic stressors of plants in the environment.

Plants show different responses to Pb stress, from visible changes to intricate cellular processes, which need further investigation to understand molecular dynamics and develop strategies like genetic modifications or phytoremediation for effective management of Pb contamination.^33^

## 6. Adaptive mechanisms of plants to Pb toxicity

Like an organism, plants too develop adaptive mechanisms against Pb toxicity which helps them in their survival in the Pb-controlled environment. The plants develop multiple adaptive responses toward the elevated level of Pb in the plants, such as plants exhibiting sophisticated biochemical pathways that detoxify Pb ions to structural modifications at the cellular level, and plants exhibit remarkable flexibility in the face of this pervasive environmental challenge.^57^

### 6.1. Pb sequestration and detoxification

The plants develop several defense mechanism approaches and strategies to counter the alleviated level of Pb. Plants have evolved mechanisms to immobilize and detoxify the Pb ions, thereby protecting cellular integrity. The absorption of Pb ions in plants takes place through their roots and translocates them inside. Further, there is an activation of special metal-binding peptides (metallothioneins and phytochelatins) which sequester the Pb ions in the cellular compartments.^141,142^ Consequently, there is a lowered of detrimental effects of Pb ion’s essential physiological processes.

### 6.2. Role of antioxidant defense systems (ADS)

The activation of the ADS is one of the major strategies to counter the harmful levels of Pb-induced oxidative stress.^143^ Antioxidants have an important role in scavenging reactive oxygen species (ROS) generated under Pb stress, which stops oxidative damage to cellular components.^144^ The ADS of plants is facilitated by superoxide dismutase (SOD), peroxidase (POD), and catalase (CAT) which are instrumental in transforming ROS into relatively benign molecules.^1^ In addition to this mechanism, several enzymatic antioxidants like ascorbic acid, glutathione, carotenoids, and tocopherols also play a significant role.^145^ Moreover, organelles like peroxisomes and mitochondria also play a valuable in ROS metabolism.^146^ ADS regulation involves the mitogen-activated protein kinase (MAPK) signaling pathway and WRKY and NAC transcription factors (TFs).^1^

#### 6.2.1. Enzymatic antioxidants (EA)

Enzymatic antioxidants are believed to be the first line of defense against Pb-induced OS in plants. SOD catalyzes the dismutation of superoxide radicals (O_2_•−) into O_2_ and hydrogen peroxide (H_2_O_2_), by avoiding the accumulation of superoxide radicals.^147^ The detoxification of the H_2_O_2_ and its conversion into H_2_O and O_2_ is performed by catalase and peroxidases which alleviate oxidative damage to cellular components. The activity and expression of EA are firmly regulated in response to exposure to Pb, along with TFs like AP2/ERF and NAC which contribute a crucial role in modulating antioxidant gene expression.^144,148^

#### 6.2.2. Non-enzymatic antioxidants

The non-enzymatic antioxidants work synergistically with enzymatic antioxidants to maintain redox homeostasis and protect the plants from OD.^149^ GSH plays a vital role as a crucial redox buffer, which participates in the scavenging of ROS and the regeneration of other antioxidants.^150^ Ascorbic acid acts as a potent antioxidant, which scavenges ROS directly and regenerates oxidized forms of other antioxidants.^151^ Some other nonenzymatic antioxidants are tocopherols (vitamin E), which also contribute majorly to the protection of cell membranes from LiPr induced by ROS.^149^

#### 6.2.3. Regulation of ADS

Due to elevated Pb levels, the ADS gets activated and regulated at multiple levels, such as transcriptional, post-transcriptional, and post-translational processes.^148^ TFs like WRKY, AP2/ERF, and MYB are involved in the regulation of antioxidant gene expression under Pb stress.^152^ In addition to this microRNAs and small peptides may control antioxidant activity through post-transcriptional and post-translational mechanisms.^153^

### 6.3. Changes in cellular transport processes

As a result of Pb toxicity, there is a disruption of essential cellular transport processes in the plants, requiring adaptive responses to sustain homeostasis. The plants start modulating the uptake, translocation, and compartmentalization of Pb ions within cells when there is a change in the membrane transporters, ion channels, and vesicular trafficking pathways.^154^

Plants could hamper the accumulation of Pb ions in specialized cellular organelles by dynamic modulation of cellular transport mechanisms while facilitating its sequestration and detoxification in the specialized storage organelles.^155^ Additionally, the interaction between cellular transport and signaling pathways permits the plants to assimilate environmental signals and coordinate adaptive reactions to Pb-induced stress.^156^ Plants activate ADS to counter the Pb-induced OS, helping in their adaptation to Pb toxicity. Investigations focus on understanding molecular mechanisms, interactions between antioxidant pathways, and developing Pb-tolerant plant varieties for phytoremediation.^58^ Understanding plant adaptive responses to Pb toxicity demonstrates their resilience and plasticity.

## 7. Influence of plant-microbe interactions on Pb tolerance

In the rhizospheric region plants and microorganisms interact with each other, which plays a crucial role in shaping the response of plants to Pb stresses, and other HMs.^82^ The interaction between the plants and microorganisms could be either a negative or a symbiotic association.^157^ Additionally, the Pb toxicity could affect the microbial species in terms of number and diversity in the rhizospheric region (Sevak et al., 2021a). The influence of the rhizosphere microbial population on the removal of Pb, and the role of microorganisms in mediating Pb immobilization, transformation, and detoxification methods has been discussed here.^158^

### 7.1. Rhizosphere microbial community and Pb remediation

The rhizosphere has a diverse and dynamic microbial population that mainly affects plant health and stress tolerance.^159^ Literature has proven that some of the bacteria (Pb-resistant bacteria) and fungi (mycorrhizal fungi) could solubilize insoluble Pb compounds, sequester Pb ions, or facilitate the uptake and translocation of Pb by plants.^160^ Besides this, microbial-mediated events such as rhizosphere acidification, metal chelation, and enzymatic degradation help in the removal of Pb from the contaminated soils.^161^ The understanding of the dynamics of the rhizosphere microbial population and their interactions with plants is very important to harness the microorganism-mediated approaches to augment the efficiency of phytoremediation and mitigate the effect of Pb contamination on the ecosystems.^162^

### 7.2. Symbiotic relationships and plant tolerance to Pb

Microorganisms and some plants have been found to undergo symbiotic association, for instance, mycorrhizal association, nitrogen-fixing bacteria (e.g., rhizobia), and plant growth-promoting rhizobacteria (PGPR).^163^ Such a positive relationship may prove to be valuable in terms of the tolerance level of Pb as such an association could facilitate nutrient acquisition, stress mitigation, and growth promotion. The mycorrhizal fungi could increase the tolerance capacity of Pb in plants, by enhancing the uptake of nutrients, plant antioxidant defenses, and modulating levels of plant hormone.^163^ The N_2_-fixing bacteria could improve the Pb-induced stress in plants by promoting the growth of plants, improving nutrient availability, and stimulating systematic resistance against plant pathogens.^164,165^ More investigations are required for a better understanding of the phenomenon involved in the symbiotic relationships and their effect on the tolerance of Pb by the plant. This could prove helpful in providing valuable information for harnessing these interactions in sustainable remediation approaches and improving plant resilience to environmental stressors.

The interaction of plants and microorganisms on Pb tolerance could represent a promising avenue for the formation of an environment-friendly and sustainable process for the elimination of Pb-contaminated environments.^58^ By understanding the dynamic of the rhizospheric microbial population and symbiotic relationship, researchers could identify the microbial species and consortia with the potential to increase the efficiency of phytoremediation and promote plant health in Pb-contaminated soils.^166^

## 8. Genetic and epigenetic regulation of Pb responses in plants

Plants apply genetic and epigenetic mechanisms to control the alleviated level of Pb. The interaction between genetic variations and epigenetic changes in plants with increased Pb toxicity is a complex mechanism.^167-169^ Genetic variations could affect the susceptibility of plants to epigenetic modifications induced by the exposure of Pb. Besides this, epigenetic changes could also influence how genetic variations will be expressed, leading to variations in plant responses to Pb stress. Plant tolerance to Pb pollution is affected by the synergistic effects of genetic variations and epigenetic changes. The understanding of the mechanism of interaction of these factors and their influence on each other is very important so that the phenomenon behind plant adaptation to environmental Pb contamination stressors can be unraveled.^168,169^ The identification of the key genes and pathways associated with plant tolerance is possible which could pave the way for developing approaches to increase plant resilience in polluted environments.

### 8.1. Genetic variation in Pb tolerance

Genetic variation acts as the basis of plant adaptation to environmental stressors such as Pb toxicity. Plant populations exhibited a spectrum of responses to Pb contamination through natural selection and genetic diversity, which may vary from susceptibility to tolerance. Plants undergo numerous genetic variations due to the increased level of Pb which could be genetic determinants of Pb tolerance, encompassing variations in genes encoding metal transporters, detoxification enzymes, and stress-responsive proteins. The confirmation of the identification of the candidate genes and alleles involved with increased Pb tolerance paved the way for targeted breeding programs and genetic engineering approaches aimed at developing Pb-resistant crop varieties.^168,170^

### 8.2. Epigenetic modifications in response to Pb stress

From the above section, it has been observed that epigenetic modifications play a valuable role in the Pb-stress-mediated response. As it helps in understanding the process involved in the expression of genes in the presence of Pb contamination. The epigenetic modifications could act as the conductors in the Pb-contaminated plants, finely tuning how genes perform in this situation. Moreover, epigenetic modifications confirm that the right genes are being activated or silenced at the right time, composing a harmonious response to environmental challenges without affecting the genetic score itself.^119,171^

DNA methylation is a common epigenetic mechanism that often leads to gene silencing. DNA methylation could influence which genes are turned on or off in response to the availability of Pb in the environment during the Pb stress.^172^ Histone modifications may affect how tightly or loosely the packaging of DNA is done, impacting gene expression.^173^ The histone modifications could alter the accessibility of certain genes, influencing the plant’s response to Pb contamination during the Pb stress plant.^174^ Small RNA-mediated gene regulation could target particular genes and either promote their expression or cause their inhibition.^175^ These small RNAs could play an important character in fine-tuning the plant’s response to the stressor under Pb stress. The epigenetic landscape of Pb stress refers to the overall pattern of epigenetic variations that takes place in plants exposed to Pb contamination.^176^ This landscape includes DNA methylation patterns, histone modifications, and small RNA activity, all of which jointly affect the mechanism of plants’ adaptation to Pb contamination. Understanding how epigenetic modifications affect gene expression is vital for decoding how plants respond to stress induced by Pb.^177^ By regulating which genes are turned on or off, epigenetic processes could regulate the plant’s capability to manage Pb toxicity and adapt to the challenging environment. As a consequence of Pb stress, there will be changes in the structure of the chromatin which will impact the ability of the plants to develop an effective response to the stressor. Besides this epigenetic modification plays a major role in the regulation of the phenotypic plasticity in plants once exposed to Pb contamination, which influences traits such as growth patterns, root development, and stress tolerance.^178^

By understanding the detailed information about the epigenetic signatures associated with Pb tolerance, researchers could identify specific epigenetic variations that are connected to a plant’s ability to tolerate Pb stress. These signatures can serve as important targets for constructing strategies to increase plant resilience to Pb toxicity. Manipulating epigenetic pathways in plants could increase resilience to Pb contamination, which offers a targeted strategy for sustainable growth in contaminated environments.

## 9. Strategies for enhancing phytoremediation efficiency

### 9.1. Remediation strategies

To date, several investigators have suggested numerous remediation strategies like phytoremediation, bioremediation, and chemical immobilization to mitigate the adverse effects of Pb pollution on soil microorganisms and soil health.^179^ Phytoremediation involves the use of plants to absorb and accumulate HMs from contaminated soil while bioremediation involves the use of microbes to degrade or transform contaminants in soil. Chemical immobilization involves the utilization of amendments, like phosphate or organic matter, to reduce the bioavailability of Pb in soil.^180^

The mechanism of Pb detoxification and tolerance includes the following events; i) sequestration, ii) phytochelatins, iii) antioxidant defense, and iv) cell wall binding. In sequestration, there is the storage of the Pb by plants in the vacuoles. There is the formation of metal-binding peptides in the phytochelatins. The antioxidant defense includes the activation of anti-oxidant systems, and in cell wall binding there is confinement of Pb to the cell walls of the plants.^154^

### 9.2. Phytoremediation

Pb is a heavy metal that accumulates in soil and adversely affects plant growth and development. Several remediation technologies could be applied to minimize the impact of Pb toxicity on plant growth.^181^ Practices like replacing the contaminated soil with clean soil followed by disposing of the polluted soil in landfills are some of the conventional approaches to remediate the soil.^182^ Such conventional approaches have limited applications due to their expensive nature so plants are preferred for the remediation of contaminated soil by phytoremediation.^183^ It is a natural biological process that degrades harmful chemicals (dyes, pesticides, etc) and transforms non-biodegradable HMs and metalloids into nontoxic forms in the soil and wastewater.^180^ Various pieces of literature have demonstrated that some of the plants are highly effective in absorbing Pb from the soil and water which may help in decontaminating the environment or ecosystem thus preventing the entry of these HMs in the food chain.^82^ Phytoremediation is capable of restoring soil ecosystems by increasing soil quality, mitigating soil erosion, and incorporating organic matter.^184^

Phytoextraction and phytostabilization are the prevalent steps used in the phytoremediation of contaminated soil. Phytoextraction is the process by which roots of the plants eliminate water-soluble HMs/Pb from the soil by absorbing and storing these elements in their aerial parts (shoots and leaves), which are then harvested and metabolized for energy and metal recycling.^184^ The effectiveness of phytoextraction in contaminated soils relies on the bioavailability of HMs for uptake by plants.^185^

**Figure 8.**
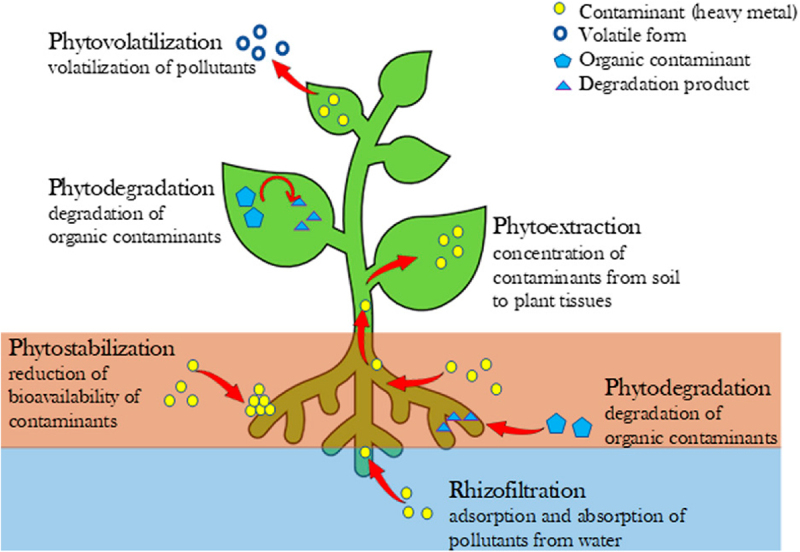
Schematic representation of different phytoremediation approaches by plants under study.^179^

Whereas in phytostabilisation plants are used to immobilize pollutants present in the soil and groundwater where the immobilization of Pb is achieved by the secretion of chemicals in the root. The deposition of the Pb in the roots takes place through absorption and adsorption. Further, instead of the dissolution of these Pb by the plants, there is a secretion of compounds by the plants that immobilize the Pb. It has been observed that the amendment of compost with contaminated soil has significantly increased the efficiency of phytostabilization, due to its ability to immobilize Pb and lower their bioavailability in soil.^186^ Attanayake et al., amended Pb-contaminated soil with composted class A biosolids (44 kg/m^2^) which resulted in a reduction of 17% Pb bioavailability compared to the untreated soil.^186^ Figure 8 exhibits different phytoremediation approaches by plants, which involve, phytovolatilization (volatilization of pollutants), phytodegradation (degradation of organic pollutants), phytoextraction (deposition of contaminants from soil to plant tissues), phytostabilization (reduction of bioavailability of pollutants), and rhizofiltration (pollutants adsorption and absorption from water).

Some of the investigations showed that rhamnolipid-enhanced *Bacillus methylotrophicus* exhibited the highest petroleum hydrocarbon removal rate from the leakage sites^53^ whereas among plants *Brassica pekinensis, Helianthus annuus*, *Vigna unguiculata*, and *Gomphrena globosa*, were also found effective in eliminating Pb from soil.^184^ In some of the investigations lime, gypsum, and phosphate were added to the soil to increase the pH resulting in a reduction in the the bioavailability of Pb to plants. Flowers like sunflowers, Indian mustard, and maize, were found very effective in the phytoremediation of Pb-polluted soils, as they can accumulate Pb in the aerial parts of the plant, which can then be harvested and eliminated from the site. The addition of ethylene diamine tetra-acetic acid (EDTA) and compost (soil amendment) can boost the effectiveness of phytoremediation by increasing both Pb absorption by plants and their biomass.^184^ EDTA treatment was shown to improve phytoextraction, by considerably increasing the total accumulation of lead in sunflowers.^184^ Seth et al., found that the addition of EDTA (500 M) increased the Pb accumulation in sunflower roots and shoots by 12% and 88%, respectively.^187^ EDTA usage has several drawbacks for instance EDTA could, negatively alter the food chain through animal exposure and metal leaching that might potentially pollute the groundwater due to its long persistence and slow breakdown in soil.^184^

Investigations revealed that compost treatment increased the growth of *Lolium italicum* and *Festuca arundinacea*, *Vigna unguiculata*, *Brassica pekinensis*, *Gomphrena globose*, and *Helianthus annuus* when grown in Pb-polluted soil and also reduced the accumulation of Pb in plants.^184^

Compost inhibits the uptake of Pb by plants by decreasing Pb mobility and bioavailability in soil. Furthermore, compost-assisted phytostabilization with cowpeas can be a potential strategy for reducing Pb exposure from contaminated soil.^184^ It can enhance Pb root accumulation by promoting root system development while lowering Pb mobility and bioavailability in soil.^184,188,189^

Biochar, which is generated from organic waste, can be mixed with soil to reduce Pb toxicity by adsorbing it onto its surface and increasing soil pH. Investigations have shown that with the utilization of phosphorus fertilizers, the toxicity of Pb was reduced for the plants as phosphorus forms an insoluble complex with Pb.^190-192^ However, the effectiveness of these remediation strategies may vary depending on the specific site conditions and the level of Pb contamination.

The above information concluded that phytoremediation is a sustainable and environment-friendly method for the removal of Pb from the soil and water, by harnessing the potential of the plant.

### 9.3. Genetic engineering approaches

Genetic engineering exhibits a huge potential for the optimization of the plants to thrive in phytoremediation endeavors. It has been found that genetic engineering enhances the features of the plant which may facilitate the phytoremediation of Pb, Pb uptake and tolerance, Pb metabolism, and biomass production. In addition to this, the genetically modified plants demonstrated enhanced metal transporters, chelating peptides, or enzymes associated with Pb removal pathways. The combination of genetic engineering with genome editing technologies such as CRISPR/Cas9 provides unprecedented precision and efficiency in modifying plant genomes could be achieved by combining procedures like genetic engineering with genome editing technologies (CRISPR/Cas9).^193^

### 9.4. Optimizing rhizosphere conditions

During the phytoremediation process, the rhizosphere plays a crucial role in mediating interactions between plants and soil pollutants. The phytoremediation efficiency of Pb could be improved by a detailed analysis of the pH of the soil, organic matter content, microbial population, and root exudates.^194^ It is possible to create a conducive environment for phytoremediation by various approaches which include promoting the proliferation of Pb-tolerant microorganisms in the rhizospheric region or increasing the root exudates secretion that helps in the uptake and metabolism of Pb.^195^ In addition to this, the integration of rhizosphere engineering approaches along with bioaugmentation or biochar amendments holds promise for increased elimination and immobilization of Pb in phytoremediation systems.^158^

### 9.5. Phytoremediation in combination with other remediation methods

**Figure 9.**
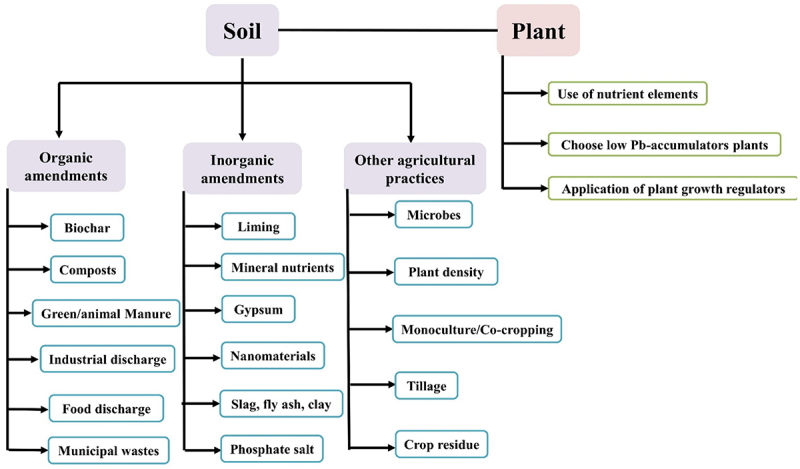
Phyto-management to reduce Pb uptake in cereals.^74^

The efficiency of Pb removal by phytoremediation could be increased by applying a synergistic effect where the phytoremediation could be clubbed with other remediation techniques.^196^ In these techniques, phytoremediation could be integrated with microbial remediation, soil amendments, and physical or chemical treatments.^197^ The maximization of contaminant removal, remediation rates, and long-term sustainability could be achieved by applying a synergistic approach. Additionally, the combination of phytoremediation and nano remediation holds promise for overcoming limitations involved with conventional phytoremediation methods for the removal of Pb.^179^ Figure 9, shows various phyto-management strategies to minimize the Pb uptake in cereals.^74^ The amendments of the soil could be either organic (biochar, composts) or inorganic (lime, gypsum, etc). Besides this, there are other agricultural practices like microbes, monocultural-cropping, etc.

### 9.6. Advantages and drawbacks

In comparison to various conventional techniques, phytoremediation is quite effective for the removal of HMs including Pb from contaminated soils. Phytoremediation has several benefits and disadvantages for the removal of Pb toxicity (Table 1).^204^

**Table 1.** Benefits and drawbacks of the phytoremediation process in Pb toxicity.

Benefits	Drawbacks	References
Could be applied to different pollutants	Requires more root surface area and depth for efficient working	^180^
Economical in comparison to conventional methods	Long-term commitment due to less production of biomass due to slow root growth in Pb-polluted soils (time-taking)	^198^
Efficient pollutant reduction	Efficiently affected by the increase in the age of plants	^199^
Less disruptive in comparison with physical elimination and chemical treatments	Plant survival under variable Pb toxicity	^200^
Eco-friendly	Variable climatic conditions adversely affect the plant’s working efficiency	^201^
Esthetically pleasing	Variable soil chemistry	^202^
Easy to monitor	Bioaccumulation of Pb in plants and its transportation in the tissue of the plants	^197^
Possibility of recovery of different metals	Availability of pollutants for primary consumers through the food chain	^203^
Recycling of metals (phytomining)	No assurance of complete removal of pollutants from soil	^204,205^

## 10. Applications and future directions

Pb-based contamination is continuously increasing in the environment so phytoremediation emerges as a promising solution for sustainable mitigation. Phytoremediation could play a diverse role in addressing Pb contamination. A lot more investigation and further studies are required for effective and enhanced phytoremediation of Pb. This technique provides a sustainable and promising approach to counter Pb pollution, by utilizing the natural capability of plants to uptake, accumulate, and detoxify pollutants from soil and water. Some of the key applications and future research directions are described below.^206^

### 10.1. Utilization of Pb-tolerant plant species

The efficacy of phytoremediation could be increased by selecting and utilizing plant species that are tolerant to Pb. Various investigations have demonstrated the natural tolerance to Pb toxicity by several plant species out of which some are hyperaccumulators and others could be metallophytes. The hyperaccumulators could accumulate higher concentrations of Pb in their tissues whereas some of the metallophytes could adapt themselves to thrive in metalliferous soils.^206^ By utilizing the unique adaptive features of these Pb-tolerant plant species, researchers could construct a tailored phytoremediation technique optimized for particular environmental contexts and contamination scenarios. In addition to this, a combined approach or synergism of molecular and physiological techniques for the identification and characterization of Pb-tolerant genotypes holds the potential for speeding up the selection and breeding of improved phytoremediation species along with the increased tolerance to Pb and remediation capability.^206^

### 10.2. Potential for sustainable remediation strategies

Phytoremediation provides a sustainable and environment-friendly technique for the removal of Pb-contaminated soil, fields, or areas. Besides this, the selected method must be low-cost, and scalable. The phytoremediation can effectively immobilize, degrade, or sequester Pb pollutants, by natural processes. This approach minimizes the requirement of expensive and energy-intensive treatment techniques. Additionally, a combination of phytoremediation with sustainable land management practices (agroforestry, riparian buffer zones, and green infrastructure) exhibits the potential for refining the health of the ecosystem, and quality of soil, and minimizing the toxic effects of Pb contamination on living beings and wildlife.^207^

### 10.3. Future research directions

The future of phytoremediation research holds enormous potential for innovation and advancement. There is a requirement for more research with a detailed molecular mechanism underlying plant responses to Pb stress. In-depth investigations are required for the complicated interactions between plants and microbial communities present in the soil contaminated by Pb and optimizing phytoremediation techniques for increased removal of Pb and long-term stability. The application of Pb-tolerant plant species, the potential for sustainable remediation approaches, and future research insights all together highlight the significance of phytoremediation as a viable and environment-friendly method for addressing Pb contamination. By utilizing the inherent potential of plants and applying interdisciplinary collaborations, phytoremediation holds promise for delivering sustainable solutions to complicated methods due to the contamination of Pb in the environment.

## 11. Conclusion

Lead toxicity is a significant environmental issue that negatively affects plant growth and development. HMs contamination, especially Pb, can deposit in plant tissues and cause oxidative stress, leading to cellular damage and reduced biomass production. However, plants have evolved a sophisticated antioxidative defense system that involves the collective action of several enzymes, organelles, and signaling pathways to mitigate the deleterious effects of Pb-induced oxidative stress. The regulation of the ADS is complex and includes several signaling pathways, including the MAPK pathway and TFs such as WRKY and NAC. The understanding of the negative effects of HMs on plant growth and their mechanisms is highly important for their uptake to develop efficient strategies to mitigate the problem. Such information could help in constructing novel phytoremediation approaches and sustainable agricultural practices that will reduce the risks of Pb toxicity in crops and ultimately promote global food security. There is a requirement and implementation of some strict regulations by Government agencies on the manufacturing industries, involved in discharging the hazardous compounds into the environment. Such strategies will help in minimizing the concentration of heavy metals in both terrestrial and aquatic environments.

## Data Availability

All the data are present in the manuscript only.
